# Changes in public perceptions and experiences of the Australian health‐care system: A decade of change

**DOI:** 10.1111/hex.13154

**Published:** 2020-11-20

**Authors:** Louise A. Ellis, Chiara Pomare, James A. Gillespie, Jo Root, James Ansell, Joanna Holt, Leanne Wells, Yvonne Tran, Jeffrey Braithwaite, Yvonne Zurynski

**Affiliations:** ^1^ Centre for Healthcare Resilience and Implementation Science Australian Institute of Health Innovation Macquarie University Sydney NSW Australia; ^2^ NHMRC Partnership Centre in Health System Sustainability Australian Institute of Health Innovation Macquarie University Sydney NSW Australia; ^3^ Consumers Health Forum of Australia Canberra ACT Australia; ^4^ Menzies Centre for Health Policy Sydney School of Public Health The University of Sydney Sydney NSW Australia

**Keywords:** Australia, consumer sentiment, health‐care system, public perception

## Abstract

**Background:**

The views and experiences of the Australian public are an important barometer of the health system. This study provides key findings about the changing views held by Australians over time regarding their individual experiences and perceptions of the overall performance of the health system.

**Methods:**

A population‐based online survey was conducted in 2018 (N = 1024). Participants were recruited through market research panels. The results were compared with previous Australian population survey data sets from 2008 (N = 1146), 2010 (N = 1201) and 2012 (N = 1200), each of which used different population samples. The survey included questions consistent with previous surveys regarding self‐reported health status, and questions about use, opinions and experiences of the health system.

**Results:**

Overall, there has been a shift in views from 2008 to 2018, with a higher proportion of respondents now viewing the Australian health‐care system more positively (*X* ^2^ (2, N = 4543) = 96.59, *P* < .001). In 2018, areas for attention continued to include the following: the need for more doctors, nurses and other health workers (29.0%); lower costs for care or Orion medicines (27.8%); more access to care (13.1%); and enhancements in residential aged care (17.3% rated these services as ‘bad’ or ‘very bad’).

**Conclusions:**

This research suggests that Australians’ perceptions of their health‐care system have significantly improved over the last decade; however, concerns have emerged over access to medicines, inadequate workforce capacity and the quality of aged care facilities. Our study highlights the value of periodically conducting public sentiment surveys to identify potential emerging health system problems.

## 1 INTRODUCTION

Population growth, ageing populations, longer life expectancy and increases in the prevalence of chronic diseases and long‐term medical conditions are creating expanded demand for health‐care services and contributing to rising health‐care costs worldwide.^1^ In Australia, as with other countries, governments are struggling to meet demands for access to new sophisticated and costly diagnostics, long‐term treatments and the growing need for more aged and hospital care,^2^ with regular claims that the increases in health‐care costs are unsustainable.^3^ This has led to calls to transform the health system—to improve efficiencies, reduce costs and continue to deliver high‐quality performance‐based care.^4^, ^5^

Surveys of public perceptions and experiences with health‐care services are important in identifying how well a country's health system is meeting the needs of its population,^6^ and can be used to leverage policy and system change. For example, the Commonwealth Fund regularly surveys public views about the United States (US) health system and health systems in 11 high‐income countries, including Australia.^7^ Results from Commonwealth Fund surveys in the United States and comparisons with international data such as these have supported significant health system reforms in the United States.^7^

Further, health‐care has a unique connection to shared national values around risk and citizenship. This is particularly true of systems providing universal coverage. Canada's universal health‐care system (Medicare) embodies the ‘social citizenship’ that distinguishes Canadians from their more market‐driven US neighbours.^8^ The British National Health Service was from its foundation treated in almost religious tones.^9^ In both these cases, health policy debate has been shaped around these enduring values.

In a similar way, the views and experiences of the Australian public have been an important barometer of the health of the Australian health system, with important implications for health‐care practice and policy. Repeated public polls on taxation and social service provision in the 1990s and early 2000s showed the high and increasing importance of health to the Australian public, and broad‐based support for Medicare's universal coverage, and for increasing expenditure on health.^10^ Public election polls have also consistently shown that ‘health and Medicare' is a highly important issue to Australian voters in every federal election since the 1970s.^11^ However, robust longitudinal studies that use validated questions about perceptions and experiences of health‐care among the adult population are rarely reported in Australia.^12^

Australia's Medicare, a national, publicly funded universal health‐care system, provides access to medical and hospital services for all Australian citizens and permanent residents.^13^ Medicare provides free or subsidized treatment by health professionals including general practitioners (GPs) and other medical specialists, and provides free public hospital treatment. Medical practitioners in private practice and private hospitals are free to charge patients what the market will bear, with a fixed subsidy from Medicare, resulting in varying patient co‐payments. A parallel Pharmaceutical Benefits Scheme provides subsidized access to most prescription medicines. Consumers can take up supplementary private health insurance to help manage some of the additional costs, which is currently held by approximately half of all Australians.^13^ Of the estimated AU$170 billion health expenditure in 2015‐2016 (representing 10% of gross domestic product [GDP]), almost 70% was funded by government sources, with 17% paid by patients through out‐of‐pocket expenses and 9% by private health insurers.^13^

Understanding what the Australian public expects and values from a contemporary health‐care system will not only complement existing surveys, such as the Australian Bureau of Statistics Australian Health Survey,^14^ but also unpack the meaning of the concerns over health and Medicare that have been a feature of Australian opinion polling. This will add additional insights into the current issues that are important to the Australian public, as well as the direction national health policy could take to address public needs and concerns.

The overarching objective of this study was to analyse a recent survey of Australians to understand their opinions of the overall performance of the health system and their individual experiences while accessing health‐care. Australian sentiment was compared longitudinally over four time points, by comparing the recent sentiment survey with Australian Health surveys conducted by the Menzies Centre for Health Policy and the Nous Group in 2008, 2010 and 2012^15^, ^16^, ^17^; allowing for the examination of a decade of change in opinions and experiences.

## 2 METHODS

### 2.1 Participants

Australian participants (aged ≥18 years) were recruited through a market research company Research Now (since rebranded as Dynata; https://www.dynata.com/), which operates several national and international panels with >11 million panellists worldwide, and over 200 000 panellists registered in Australia. Research Now panellists have opted to participate in online survey research; in exchange for their participation, panellists receive small rewards, including cash, items or reward points.

For this study, Research Now was contracted for 1000 completed surveys based on representative quotas for age, gender and geographical location. A sample size of 1000 was sought, to be in keeping with previous Menzies‐Nous surveys, and which was deemed to be large enough to detect group differences. The sample for this study was randomly selected from the Research Now general population panel of Australians 18 years and older. To be representative of the general population, the sample was deployed in batches, controlling for age, gender and geographical location based on the 2016 Australian Bureau of Statistics (ABS) census data.^18^ Potential participants were invited to take part via email. Informed consent was provided through the opt‐in process and the action of choosing to participate in a given survey. Participants were provided with the contact details of the primary investigator (YZ) in the event that they had questions or wanted further information about the study. No incentive was offered by the researchers; however, participants were paid a small fee (AUD$1.50) by Research Now for completing the survey. Ethical approval was provided by the Macquarie University Human Research Ethics Committee (Ref no: 5201836705403).

### 2.2 Survey

The survey was conducted from 29 November to 14 December 2018 and included a total of 39 questions, with selected items from the National Health Survey,^14^ the three biannual Menzies‐Nous Australian Health Surveys^15^, ^16^, ^17^ and items developed by the authors. The survey had good overall internal consistency reliability with Cronbach's alpha of 0.75. The Menzies‐Nous surveys used computer‐assisted telephone interviewing (CATI) methods with random digit dialling (RDD). The 2012 survey supplemented this with RDD of mobile phones to allow for the rapid change in communication technology, especially among younger people. At each time point, the Menzies‐Nous surveys recruited separate representative population samples. The 2018 survey items reported in this paper only include questions that were consistent with the Menzies‐Nous surveys to enable longitudinal comparisons: self‐reported health status, and questions about participants’ use, opinions and experiences of the health system. They are described in further detail below (also see Appendix 1 for the full survey). The results of the remaining items will be published elsewhere.

### 2.3 Self‐reported health status

Based on the previous Menzies‐Nous surveys,^15^, ^16^, ^17^ participants were asked how they would describe their own health. This item was rated on a 5‐point Likert scale (1 = excellent to 5 = poor).

### 2.4 General opinions regarding the quality of health‐care services

Participants were asked to provide their general opinion regarding the quality of a range of health‐care providers on a 5‐point Likert scale (1 = the service is very bad to 5 = the service is excellent). This question was included in two of the previous Menzies‐Nous surveys (2012, 2010), but not in 2008.

### 2.5 Visits to general practice

To assess the extent of GP use, participants were asked when they go to their GP, do they: 1 = always try to see the same GP; 2 = always go to the same GP practice but see different doctors; or 3 = go to a GP practice and see whichever doctor is available at the time. This question was included in all three previous Menzies‐Nous surveys.^15^, ^16^, ^17^

### 2.6 Overall views towards the Australian health‐care system and areas for improvement

Participants were asked to express their overall views of the Australia health‐care system on a 3‐point scale (1 = on the whole, the system works pretty well and only minor changes are needed to make it work better to 3 = our health‐care system has so much wrong with it that we need to completely rebuild it). This question was included in all three previous Menzies‐Nous surveys.^15^, ^16^, ^17^ Participants were also asked to identify the areas of the health‐care system they thought needed the most improvement. Equivalent data to this question were only available from the 2012 Menzies‐Nous survey.

### 2.7 Confidence in the Australian health‐care system

To assess confidence in key areas, participants were asked if they were to become seriously ill, how confident would they be that they would: get quality and safe medical care; receive the most effective medication; receive the best medical technology; and be able to afford the care needed. Participants provided responses to each of these four areas on a 4‐point Likert scale (1 = very confident to 4 = not at all confident). This question was included in all three of the previous Menzies‐Nous surveys.^15^, ^16^, ^17^

### 2.8 Data analysis

Survey data collected in 2018 were post‐weighted by age, sex and state to reflect population distribution according to the Australian Bureau of Statistics (ABS) demographic statistics of June 2018.^19^ Previous raw data from the Menzies‐Nous Australian Health Surveys were made accessible to the research team, which we also post‐weighted by age, sex and state according to the ABS demographic statistics of June of the respective year.^20^, ^21^, ^22^ Each of the four surveys was post‐weighted through a survey raking technique using the anesrake package in R.^23^

Survey data were analysed using IBM SPSS Statistics Version 25.0.^24^ Comparisons across the four surveys were only made where questions were identical. Linear regression was used to examine relationships between age groups, gender, location and survey year for each of the dependent variables with five or more levels (i.e., self‐reported health status and general opinions regarding the quality of health‐care services).^25^ Four sets of dummy variables were examined for each of the categorical measures of age and survey year. For brevity, the results for all of these dummy variables are not presented here, but are available on request. Chi‐square (*χ* ^2^) analysis was used to examine categorical dependent variables for which there was less than five levels (i.e., visits to general practice, overall views towards the Australian health‐care system and areas for improvement, and confidence in the Australian health‐care system). Due to the large number of tests, a conservative *P* value of .001 was used for statistical significance.

## 3 RESULTS

### 3.1 Characteristics

In total, 1024 Australians participated in the 2018 Australian sentiment survey. Research Now did not provide the research team with the total number of contacts made to result in the final 1024 respondents. Participants were aged between 18 and 88 years (M = 46.6; SD = 17.2), with 51.0% of the sample being female. Almost half of the respondents reported that their average weekly household income after tax was between $500 and $1499 (n = 491; 47.9%), and less than $500 for 20.6% (n = 211), which is broadly consistent with national ABS data from the Survey of Income and Housing 2017/2018.^26^

Unweighted and weighted participant demographics are presented in Table 1, along with a comparison of participant demographics from the three previous Menzies‐Nous Australian Health Surveys.^15^, ^16^, ^17^ As shown, our post‐weights were successful in creating four data sets that were appropriate for comparison, taking into account differences in demographics.

**TABLE 1:** Study participant characteristics across four surveys

Characteristics	2018 *n* ^a^ (%)^b^	2012 *n* ^a^ (%)^b^	2010 *n* ^a^ (%)^b^	2008 *n* ^a^ (%)^b^
Overall	1024	1200	1201	1146
Gender				
Male	432 (49.0%)	539 (49.0%)	540 (49.0%)	420 (49.0%)
Female	592 (51.0%)	661 (51.0%)	661 (51.0%)	726 (51.0%)
Age				
18‐24 y	68 (12.0%)	116 (12.0%)	104 (12.0%)	72 (12.1%)
25‐44 y	352 (37.0%)	379 (37.0%)	397 (38.0%)	332 (38.4%)
45‐64 y	383 (32.0%)	479 (33.0%)	504 (33.0%)	492 (34.3%)
65 y+	221 (19.0%)	226 (18.0%)	196 (17.0%)	242 (15.2%)
State^c^				
ACT	9 (2.0%)	20 (2.0%)	20 (1.7%)	34 (2.0%)
NSW	330 (32.0%)	396 (32.0%)	397 (33.2%)	360 (33.0%)
NT	2 (1.0%)	11 (1.0%)	11 (1.0%)	17 (1.0%)
Qld	218 (20.0%)	233 (20.0%)	233 (19.2%)	233 (20.0%)
SA	83 (7.0%)	92 (7.0%)	92 (7.5%)	99 (7.0%)
Tas	22 (2.0%)	29 (2.0%)	29 (2.2%)	5 (2.0%)
Vic	262 (26.0%)	301 (25.0%)	301 (25.1%)	254 (25.0%)
WA	98 (10.0%)	118 (11.0%)	118 (10.1%)	143 (10.0%)
Location				
Capital city	654 (65.6%)	772 (65.0%)	773 (61.5%)	637 (54.9%)
Regional/remote	370 (34.4%)	428 (35.0%)	428 (38.5%)	508 (45.1%)

^a^ Unweighted.

^b^ Weighted for age, sex and state.

^c^ All data not available for past surveys.

### 3.2 Self‐rated health status

In 2018, the majority of Australians rated their own health as either good (n = 337, 37.0%) or better (n = 414, 40.5%). However, health status ratings in 2018 were found to be significantly lower than previous years (*P* < .001), with an average of 55.9% (n = 1982) of Australian rating their own health as very good or excellent across the previous Menzies‐Nous surveys. Across the four surveys, younger Australians (aged 18‐44 years) rated their health significantly higher than older Australians (aged 45 to 65+ years; *P* < .001), and Australians in cities rated their health significantly higher than Australians in rural or remote regions (*P* < .001). No significant differences were found for gender (Table 2). The regression results presented in Table 2 summarize the results from key dummy variables showing: age differences between younger participants (aged 18‐44 years; coded 0) and older participants (aged 45 to 65+ years; coded 1); and differences between the previous Menzies‐Nous surveys (coded 0) and the 2018 survey (coded 1).

**TABLE 2:** Regression analysis for variables predicting self‐rated health status and consumer satisfaction with health‐care services

	Age	Gender	Location	Survey
Self‐rated health status				
*β*	−0.13	0.03	−0.06	−0.13
*T*	−8.71*	2.26	−4.04*	−8.93*
95% CI	−0.41 to −0.26	0.01 to 0.13	−0.19 to −0.07	−0.39 to −0.25
Satisfaction with health‐care services				
Pharmacist or chemist				
*β*	0.06	−0.01	0.01	−0.04
*t*	2.97	−0.54	0.53	−1.80
95% CI	0.05 to 0.22	−0.09 to 0.05	−0.05 to 0.09	−0.13 to 0.01
GP				
*β*	0.06	0.001	0.003	0.11
*t*	2.58	0.03	0.15	5.01*
95% CI	0.03 to 0.23	−0.08 to 0.08	−0.08 to 0.09	0.12 to 0.28
Specialist doctor				
*β*	0.05	−0.01	0.02	0.06
*t*	2.19	−0.33	0.91	2.81
95% CI	0.01 to 0.25	−0.11 to 0.08	−0.05 to 0.14	0.04 to 0.23
Dentist				
*β*	0.02	−0.01	0.02	0.16
*t*	0.91	−0.59	1.11	7.85*
95% CI	−0.07 to 0.18	−0.13 to 0.07	−0.04 to 0.16	0.29 to 0.48
Private hospital				
*β*	−0.001	−0.03	0.02	0.03
*t*	−0.05	−1.52	0.70	1.31
95% CI	−0.12 to 0.12	−0.17 to 0.02	−0.07 to 0.14	−0.03 to 0.16
Public hospital				
*β*	0.02	−0.02	0.02	0.15
*t*	0.97	−0.911	0.94	7.35*
95% CI	−0.06 to 0.18	−0.14 to 0.05	−0.05 to 0.15	0.26 to 0.46
Allied health provider				
*β*	0.02	−0.03	−0.02	0.07
*t*	0.86	−1.21	−0.85	3.17
95% CI	−0.07 to 0.17	−0.16 to 0.04	−0.14 to 0.06	0.06 to 0.25
Mental health provider				
*β*	0.05	−0.02	0.01	0.01
*t*	2.27	−0.75	0.46	0.57
95% CI	0.03 to 0.35	−0.18 to 0.08	−0.10 to 0.17	−0.09 to 0.17
Community care				
*β*	0.02	−0.03	0.03	0.04
*t*	1.11	−1.18	1.60	1.79
95% CI	−0.06 to 0.23	−0.19 to 0.05	−0.02 to 0.22	−0.01 to 0.22
Aged care				
*β*	0.03	−0.06	−0.02	0.04
*t*	1.21	−2.81	−0.86	1.94
95% CI	−0.06 to 0.27	−0.32 to −0.06	−0.20 to 0.08	−0.002 to 0.26

* *P* < .001; age group (18‐44 y= 0, 45‐65+ y = 1); gender (male = 0, female = 1); location (0 = city, 1 = rural/remote); survey year (2008, 2010, 2012 = 0, 2018 = 1).

### 3.3 General opinions regarding the quality of health‐care services provided in Australia

**FIGURE 1:**
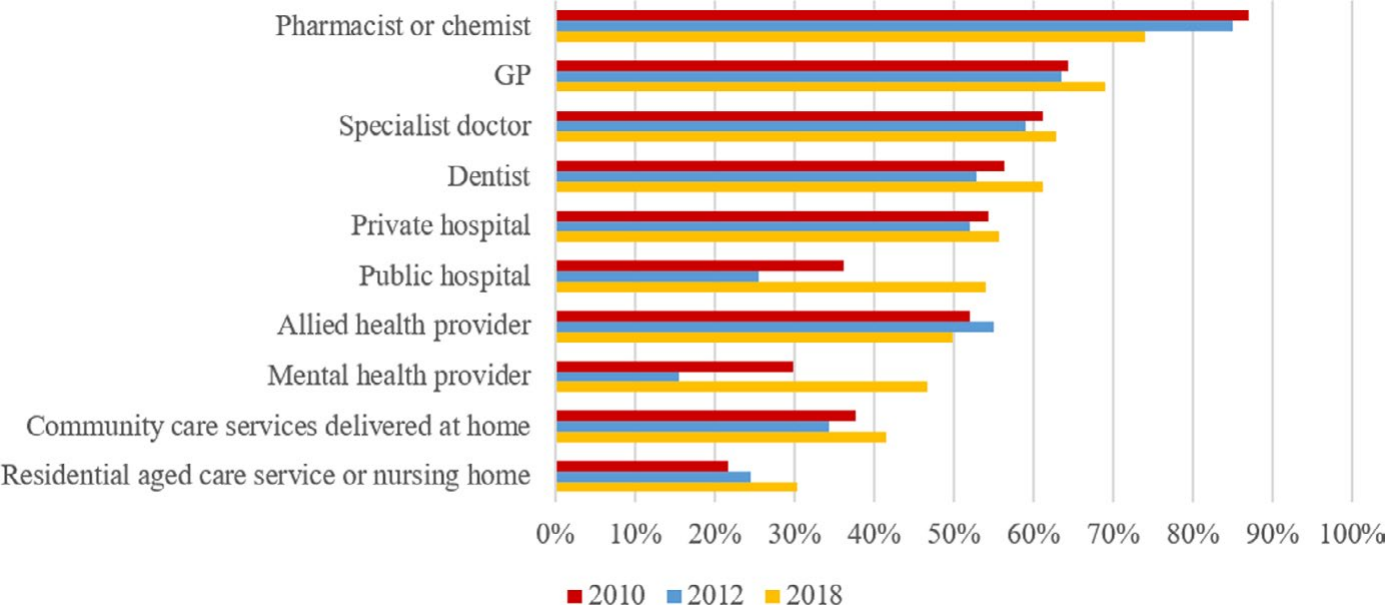
Opinions about the quality of health‐care services rated as good or excellent over time

Respondents were asked to provide their opinion on the quality of a range of health‐care services (see Figure 1 for comparisons over time). In 2018, Australians reported greatest approval for the services provided by pharmacists/chemists and GPs, with 74.0% (n = 758) and 69.1% (n = 708) rating their services as good to excellent, respectively. Residential aged care services were rated the lowest in 2018, with 17.3% (n = 177) of Australians rating the services as bad or very bad. In 2018, ratings were significantly more favourable than the previous Menzies‐Nous surveys for public hospitals (*P* < .001), GPs (*P* < .001) and dentists (*P* < .001). No other significant differences were found based on survey year, age, gender or geographical location (Table 2).

### 3.4 Visits to general practice

In 2018, the majority of participants (n = 761, 74.3%) reported that they always try to see the same GP. This was significantly higher than in previous years (2012, n = 770, 64.2%; 2010, n = 797, 66.4%; 2008, n = 619, 54.0%), as determined by a chi‐square analysis (*X* ^2^ (1, N = 4571) = 229.15, *P* < .001). No other significant differences were found for this question based on age, gender or geographical location.

### 3.5 Overall views towards the health‐care system

**FIGURE 2:**
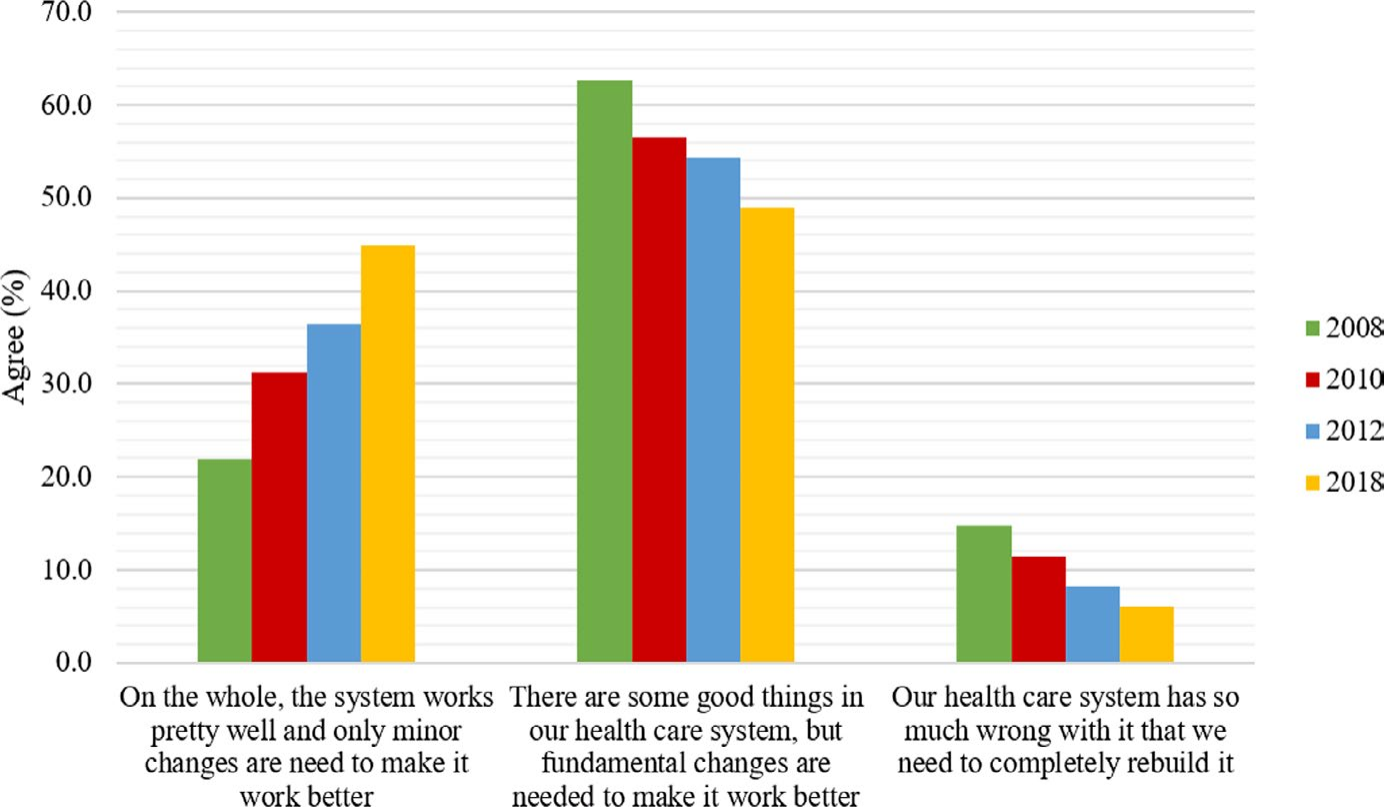
Australians’ views of the health‐care system

In 2018, almost half of Australian participants reported that ‘there are some good things in the Australian health‐care system, but fundamental changes are needed to make it work better’ (n = 502, 49.0%). However, there has been a shift in views over the past 10 years, with a higher proportion of respondents now viewing the Australian health‐care system more positively (*X* ^2^ (2, N = 4543) = 96.59, *P* < .001; Figure 2). In 2018, close to half of participants (n = 469, 45.8%) identified that the health ‘system works pretty well and only minor changes are needed to make it work better’, up from just 30% across the previous Menzies‐Nous surveys. Across the four surveys, overall views in the health‐care system differed significantly by age groups. Trends were consistent, showing that Australians aged 25‐64 years were more likely to identify a need for fundamental changes to be made to the health‐care system compared with those in the youngest and oldest age groups (18‐24 years and 65+) (*X* ^2^ (2, N = 4535) = 53.95, *P* = <.001). Across the four surveys, a higher proportion of Australians living in rural or remote regions identified the need to completely rebuild the health‐care system (n = 243, 14.2%) compared with Australians living in cities (n = 245, 8.6%) (*X* ^2^ (2, N = 4542) = 49.25, *P* = <.001).

### 3.6 Areas for improvement

In 2018, respondents reported that the greatest improvement needed to the health‐care system is: the need for more doctors, nurses and other health workers (n = 297, 29.0%); reductions in the cost of care or medicines (n = 284, 27.8%); and getting better access to care (n = 134, 13.1%). These desired areas of improvement were also reported in the 2012 Menzies‐Nous survey; however, there were statistically significant differences between 2012 and 2018 (*X* ^2^ (2, N = 1766) = 42.07, *P* < .001). In 2018, Australians rated a higher need to improve the cost of care or medicines compared with 2012 (27.8% vs 23.1%) and a significantly lower need for more doctors, nurses and other health workers, compared with preferences reported in 2012 (29.0% vs 49.1%). There was no statistically significant difference in the perception that health care should be improved by getting better access to care in 2012 (n = 185, 15.4%) compared with 2018. No comparable data were available for 2010 and 2008.

### 3.7 Confidence in the Australian health‐care system

In 2018, over 80% of respondents reported that they were ‘somewhat’ or ‘very confident’ that upon becoming seriously ill, they would receive: quality and safe medical care (n = 898, 87.7%); the most effective medication (n = 879, 85.8%); and the best medical technology (n = 843, 82.4%). However, less than two‐thirds of respondents expressed confidence that they would be able to afford the care needed (n = 641, 62.7%).

This was similar to sentiments reported by Australians across the previous Menzies‐Nous surveys, where most participants reported being ‘somewhat’ or ‘very confident’ to get quality and safe medical care (2012, n = 1060, 89.2%; 2010, n = 1077, 90.2%; and 2008, n = 1005, 88.5%) and receive the most effective medication (2012, n = 1052, 88.6%; 2010, n = 1051, 88.9%; and 2008, n = 976, 88.2%)

However, there was a statistically significant difference regarding confidence in receiving best medical technology and being able to afford the care needed, with Australians in the previous Menzies‐Nous surveys being significantly more confident in receiving the best technology (2012, n = 1033, 87.2%; 2010, n = 1036, 87.3%; and 2008, n = 954, 85.0%) (*X* ^2^ (3, N = 4516) = 28.12, *P* < .001) and affording care (2012, n = 875, 73.6%; 2010, n = 855, 72.5%; and 2008, n = 815, 72.3%) (n = 2545, 72.8%; *X* ^2^ (3, N = 4519) = 79.32, *P* < .001) compared with 2018. No other significant differences were found based on age, gender or geographical location.

## 4 DISCUSSION

This study provides unique insights into the views held by Australians about their individual experience and the overall performance of the health system. Overall, there are predominantly positive views towards the Australian health system and these have improved over the past decade. Almost half of Australians view their health‐care system positively, and this is a significant improvement from only 30% in 2012.^16^ In 2018, public sentiment was significantly more favourable towards public hospitals, GPs, and dental services than previously reported,^15^, ^16^ reflecting improved satisfaction with these services.

In 2018, three in four Australians reported that they always try to see the same GP; this is also up significantly from the previous Menzies‐Nous surveys and reinforces its findings that Australians are comfortable with a consistent relationship with a single primary care practice.^15^, ^16^, ^17^, ^27^ This is a substantial issue in current health policy. Recent policy proposals have advocated a move towards the New Zealand model of voluntary patient registration on the grounds that ‘having a regular GP is beneficial for patient outcomes, patient experience and value for the system.’^28^ This study points to over a decade of survey research that shows this would be welcomed by patients, despite rejection by some GPs who view registration as ‘giving a loaded gun to governments’.^29^

In 2012 and 2018, close to 90% of respondents were confident that they would receive quality and safe care on becoming ill, signalling high levels of confidence in the Australian health system. However, less than two‐thirds of respondents expressed confidence that they would be able to afford the needed care. The concern among Australians regarding the affordability of health care is consistent with past research,^10^ and an area that demands further exploration. Our findings of increasing concerns about affordability across the decade of surveys also concur with recent reports regarding increasing out‐of‐pocket expenses,^30^ increasing private health insurance premiums and less value for money of private health insurance.^31^ Despite increasing costs of health care to consumers and government, health outcomes have not improved in Australia over the last 10 years; for example, potentially preventable hospitalizations remained static and adverse events in hospital increased.^32^ Our survey did not specifically question respondents about care quality, health outcomes and perceived value of care accessed, and this should be considered for inclusion in future surveys. Such data may further support reform to move health system performance towards value‐based, affordable health care.^33^, ^34^

There were several other key areas of improvement identified by the Australian public, including the following: the need for more doctors, nurses and other health workers; a lowering of the cost of care or medicines; and securing better access to care. For Australians living in rural and remote regions, there was a significantly greater need to completely rebuild the health‐care system compared with Australians living in cities (14.2% vs 8.6%). These identified areas of improvement have remained consistent concerns for at least five years and signal needed improvements in the health system to meet public needs and experiences.

Among all health services, residential aged care services were rated most poorly in 2018, with fewer than one in three Australians rating the services as good to very good. This has been a consistent concern raised over the past 10 years, highlighting the on‐going need for improvement in the aged care sector. The concerns of the Australian public have continued despite several reforms to improve the aged care system.^35^ The Royal Commission into Aged Care Quality and Safety was established in 2018 to investigate the quality and safety of care provided to older people receiving aged care services at home and in residential aged care facilities.^35^ Public perceptions expressed in our survey are consistent with the Royal Commission's recent report that described the Australian aged care system as fragmented, poorly managed and underfunded.^36^ Furthermore, the recent COVID‐19 crisis has highlighted the vulnerability of the sectors with several aged care facilities designated as outbreak hot spots, with one facility reporting over 16 deaths.^37^

### 4.1 Strengths and limitations

A unique strength of this study is that it compares the views of Australians across four time points, summarizing a decade of change in opinions about the health system. Such data are rarely available elsewhere. The samples were representative for age groups, gender and geographical distribution across the four time points, and the sample sizes were large enough to support statistical confidence and power. Health consumer representatives from the Consumers Health Forum of Australia participated in the co‐design and deployment of this survey, and provided vital advice about analysis and interpretation of results. Collaboration with the Menzies Centre for Health Policy provided further input into the co‐design and access to longitudinal data for comparison with the 2018 survey. However, there were limitations to the comparisons over time, as not all questions were asked at all four time points. Further, all the Menzies‐Nous surveys collected data via CATI methods.^16^ This change of methods reflects rapid shifts in technology—Web‐based panel surveys emerged with the decline in fixed telephones. CATI methods have also been criticized as susceptible to underreporting of sensitive information.^38^, ^39^ On the other hand, Web‐based surveys have problems with bias towards respondents with access and familiarity with computer or smartphone technologies and the payment of members may also cause selection bias.^40^ Further, to avoid issues with survey fatigue, the number of survey questions was kept to a minimum and did not include questions about cultural background (e.g., country of birth) and living situation (e.g., live alone or with others). Given that there is evidence to suggest that cultural background and household situation can influence perceptions and experiences with the health system,^41^, ^42^ we will aim to include such questions in similar future surveys. Finally, we were unable to establish a survey response rate because of the sampling process applied to an established panel.

### 4.2 Implications and conclusions

Our results are important indicators of the functioning of the health system as viewed through the eyes of the most important stakeholder groups—the population and health consumers served by the system. We provide important information that should be taken into account by policymakers, health services and health providers when developing health policy to support and improve health system performance. Our study highlights the value of conducting surveys of public sentiment periodically over time, as it shows continuities in public opinion that may reflect structural problems in the system. While future surveys should be conducted to gauge changes in opinions, to support future policy and advocacy for health system improvement regular polling using comparable questions will enrich the emerging picture of the Australian values around health‐care.

Comparison across these surveys has shown that Australians’ perceptions of their health‐care system has significantly improved over the last decade. Problem areas have been identified across the surveys, including the need for more doctors, nurses and other health workers. Cost barriers have become more of an issue across the decade, particularly barriers to access to care and medicines, along with rising concern over the quality of residential aged care services.

## CONFLICT OF INTEREST

None to declare.

## AUTHOR CONTRIBUTIONS

LAE, JAG, JR, JH, LW and YZ designed the survey; JA, LW and JR organized data collection; LAE, YT, CP and JAG undertook data analysis and interpretation of results; all authors contributed to the writing of the manuscript.

## Data Availability

The data that support the findings of this study are available on request from the corresponding author. The data are not publicly available due to privacy or ethical restrictions.

## Appendix 1

### Public Sentiment Survey Instrument

**Table:**

Qu #	Area/sub area	Question text	Answer options/format
1	Demographics/gender	What is your gender?	*Select one:* ‐ Male ‐ Female ‐ Other ‐ Prefer not to answer
2	Demographics/age	In what year were you born? (enter 4‐digit birth year; for example 1976)	Open ended four digit numerical
3	Demographics/location	What is your post code? (enter a 4‐digit postcode; for example 2000)	Open ended four digit numerical
4	Demographics/educational attainment	What is the highest level of education that you have completed? Note. If you are currently studying, but have not completed a qualification, please answer with the highest level you have completed.	*Select one* ‐ Less than year 12 ‐ Year 12 or equivalent ‐ TAFE qualification, technical/trade certificate or diploma ‐ Bachelors degree ‐ Postgraduate degree or higher ‐ Don't know
5	Demographics/socio economic status	What is your household income per week after tax? Note. Household income is the amount of money coming into the household from all sources including employment, government support schemes (eg, Centrelink payments, unemployment benefits etc, for all household members)	*Select one* ‐ Under $500 per week ‐ $500 to $1499 per week ‐ $1500 to $2499 per week ‐ $2500 or more per week
6	Demographics/cost tolerance	In the last 12 mo, did any of the following happen to you because of a shortage of money?	*Select as many as apply* ‐ Could not pay the electricity, gas or telephone bills on time ‐ Could not pay the mortgage or rent on time ‐ Pawned or sold something ‐ Went without meals ‐ Was unable to heat home ‐ Asked for financial help from friends or family ‐ Asked for help from welfare/community organisations ‐ Could not pay for health care or medicine you needed ‐ None of these *[Note. This option was only able to be selected if none of the above list was selected]*
7	Demographics/access to subsidized health‐care costs	Do you have a Medicare Card?	*Select one* ‐ Yes ‐ No
8	Private health insurance/do they have it?	Do you have private health insurance?	*Select one* ‐ Yes ‐ No ‐ Don't know
9	Private health insurance/what it covers	Which best describe what your private health insurance covers?	*Select one* ‐ Hospital cover only ‐ Extras treatment cover (general or ancillary) only ‐ Hospital and Extras ‐ Don't know
10	Demographics/access to subsidized health‐care costs	Do you have a health care concession card, eg, Health Care Card (including low income cards, foster carer cards); Commonwealth Seniors Health Card; Pensioner Concession card; or a Veterans’ Health Card (Gold or White health care card)	*Select one* ‐ Yes ‐ No
11	Self‐rated health	In general, how would you describe your own health?	*Select one* ‐ Excellent ‐ Very good ‐ Good ‐ Fair ‐ Poor
12	Health concerns	At the moment, do you have any of the chronic illnesses listed, that have lasted, or are likely to last, for six months or more? If so, please select which ones	*Select as many as apply* ‐ Arthritis ‐ Asthma ‐ Back pain or back problems ‐ Cancers (such as lung and colorectal cancer) ‐ Cardiovascular disease (such as coronary heart disease and stroke) ‐ Chronic obstructive pulmonary disease ‐ Diabetes ‐ Mental disorders (such as depression or anxiety) ‐ No, none of the above
13	Experience of the health system/experience of using specific types of services	Please indicate which of the following health services you have used in the last 12 mo	*Select as many as apply* ‐ A public hospital ‐ A private hospital ‐ A GP ‐ A nurse who works in a general practice ‐ A dentist or dental services ‐ A pharmacist ‐ A specialist doctor outside hospital (eg a cardiologist, surgeon, psychiatrist) ‐ A counsellor or psychologist ‐ A community‐based healthcare service ‐ An allied health service provider, such as a physiotherapist or dietician** ‐ An alternative therapies practitioner eg acupuncture, naturopathy etc
14	Experience of the health system/satisfaction with services used	Thinking about the services that you have used in the last 12 mo, how satisfied were you with the most recent experience of using them?	*Likert scale—1 (not at all satisfied)‐5 (entirely satisfied)*. ‐ A public hospital ‐ A private hospital ‐ A GP ‐ A nurse who works in a general practice ‐ A dentist or dental services ‐ A pharmacist ‐ A specialist doctor outside hospital (eg a cardiologist, surgeon, psychiatrist) ‐ A counsellor or psychologist ‐ A community care service ‐ An allied health service provider, such as a physiotherapist or dietician ‐ An alternative therapies practitioner eg acupuncture, naturopathy etc
15	Experience of the health system/overall satisfaction	Overall, how satisfied are you with the quality of health care you have received during the last 12 mo? Would you say that you are:	*Select one* ‐ Completely satisfied ‐ Very satisfied ‐ Somewhat satisfied ‐ Not all satisfied ‐ Haven't received healthcare in the last 12 mo ‐ Don't know
16	Experience of the health system/experience of using specific modalities of service	In the last 12 mo, have you accessed healthcare by means other than attending an appointment with a health professional or attending hospital (eg via someone coming to your home, via telephone or videoconferencing or using a helpline such as Lifeline)?	*Select one* ‐ Yes ‐ No
17	Experience of the health system/experience of using other modes of care	Which of the following modes of accessing healthcare have you used in the last 12 mo?	*Select as many as apply* ‐ Consultation with any health professional via telephone or video conferencing ‐ Telephone advice line (eg Healthdirect, Lifeline, Beyondblue) ‐ Email or webchat helpline or advice line (eg, headspace online) ‐ A GP came to my home ‐ A health professional other than a GP came to my home ‐ An internet or phone app that answered specific healthcare questions ‐ An internet or phone app through which I tracked my behaviours or which provided specific health advice (eg, physical activity, blood pressure, diet)
18	Experience of the health system/experience of using other modes of care	Thinking about the different modes of healthcare you have used in the last 12 mo how satisfied have you been with them?	*Likert scale—1 (not at all satisfied), and 5 (entirely satisfied)*. ‐ Consultation with any health professional via telephone or video conferencing ‐ Telephone advice line (eg Healthdirect, Lifeline, Beyondblue) ‐ Email or webchat helpline or advice line (eg, headspace online) ‐ A GP came to my home ‐ A health professional other than a GP came to my home ‐ An internet or phone app that answered specific healthcare questions ‐ An internet or phone app through which I tracked my behaviours or which provided specific health advice (eg, physical activity, blood pressure, diet)
19	Experience of the health system/interest in using other modes of care	If you were offered any of the following modes of healthcare how likely would you be to use them?	*Likert scale—1 (not interested at all)‐5 (very interested)* ‐ Consultation with any health professional via telephone or video conferencing ‐ Telephone advice line (eg Healthdirect, Lifeline, Beyondblue) ‐ Email or webchat helpline or advice line (eg, headspace online) ‐ A GP came to my home ‐ A health professional other than a GP came to my home ‐ An internet or phone app that answered specific healthcare questions ‐ An internet or phone app through which I tracked my behaviours or which provided specific health advice (eg, physical activity, blood pressure, diet)
20	Experience of the health system/help seeking	If you need to seek help for a health problem where would you usually begin?	*Select one* ‐ Search the internet ‐ Speak with friends or family ‐ See a GP ‐ See a pharmacist ‐ See a health professional specific to the health problem (eg physiotherapist, psychologist) ‐ See an alternative health practitioner (eg naturopath, herbalist) ‐ Other (please specify)
21	Experience of the health system/help seeking online	When online searching for information about a health problem, what would you usually do?	*Select one* ‐ Use a search engine to search for information specific to your circumstances (eg, Google) ‐ Deliberately access a health specific website (e.g health direct, WebMD) ‐ Deliberately access a non‐health website (eg Facebook) ‐ Follow a link you came across on another website (eg social media) ‐ Other (please specify)
22	Experience of the health system/experience of primary care	When you go to the GP do you:	*Select one* ‐ Always try to see the same GP ‐ Always go to the same GP practice but I see different doctors ‐ Go to a GP practice and I see whichever doctor is available at the time ‐ Often go to a different GP or GP practice
23	Preferred models for primary care/ continuity of care	How important are the following points when you need to see a GP?	*Likert scale‐ 1 (not at all important) ‐ 5 (very important)*. ‐ It's important to me to see the same doctor every time I visit the GP practice even if I have to pay extra ‐ It's important that I always go the same GP practice as it is near where I live ‐ It's important that my doctor bulk‐bills through Medicare so I don't have to pay any additional fees ‐ It's important that I can get in to see my GP on the day that I’m sick ‐ It's important that I can choose to go to any GP practice or clinic I want when I’m sick
24	Access and barriers/access to care outside of hours	How easy or difficult is it for you to get medical care in the evenings, on weekends, or holidays without going to hospital emergency department. Is it….	*Select one* ‐ Very easy ‐ Somewhat easy ‐ Somewhat difficult ‐ Very difficult ‐ Never needed care in the evenings, weekends or holidays ‐ Don't know
25	Access and barriers/things they didn't do	During the last 12 mo, was there a time when you:	*Select as many as apply* ‐ Did not fill a prescription for medicine, or skipped doses ‐ Had a medical problem but did not visit a doctor ‐ Skipped a medical test, treatment, or follow up that was recommended by a doctor ‐ Did not visit a dentist when needed
26	Access and barriers/cost, transport and distance, timeliness of service	Of the actions you identified that you did not do in the last 12 mo, what was the reason that you did not?	*[Note this question was presented individually for as many of the reasons as they selected above]*. ‐ I could not afford it ‐ I was too busy with other commitments (eg work, school or family) ‐ I was not able to travel to the place that I needed to access it ‐ The service was not available at a time that I could access it (eg outside of business hours, or on a weekend) ‐ I felt ashamed, nervous or embarrassed about the service I needed to access ‐ I decided the service was unnecessary ‐ Another reason not specified above
27	Health literacy/navigating the health system and ability to actively engage with health‐care providers	Please indicate how difficult or easy the following tasks are for you now ‐ Make sure that healthcare providers understand your problems properly ‐ Feel able to discuss your health concerns with a healthcare provider ‐ Have good discussions about your health with doctors ‐ Discuss things with healthcare providers until you understand all you need to do ‐ Ask healthcare providers questions to get the health information you need ‐ Find the right healthcare ‐ Get to see the healthcare providers I need to ‐ Decide which healthcare provider you need to see ‐ Make sure you find the right place to get the healthcare you need ‐ Find out which healthcare services you are entitled to ‐ Work out what is the best care for you	*Select one per item listed (matrix question)* ‐ Cannot do or always difficult ‐ Usually difficult ‐ Sometimes difficult ‐ Usually easy ‐ Always easy
28	Views about the health system/high‐level opinions	Which of the following statements comes closest to expressing your overall view of the health care system in Australia?	*Select one* ‐ On the whole, the system works pretty well and only minor changes are needed to make it work better; or ‐ There are some good things in our health care system, but fundamental changes are needed to make it work better; or ‐ Our health care system has so much wrong with it that we need to completely rebuild it
29	Views about the health system/opinion about spending	Do you think the amount governments spends on the health of Australians is:	*Select one* ‐ Too low ‐ About the right amount ‐ Too high ‐ Don't know
30	Views about the health system/preparedness to personally contribute more $	Would you be prepared to contribute more than the current 2% Medicare levy on your taxable income if this meant you paid more tax overall?	*Select one* ‐ Yes ‐ No ‐ Don't know
31	Views about the health system/what can be improved	Which one of the following areas of the health system do you think needs the most improvement?	*Select one* ‐ Getting better access to care ‐ The cost of care or medicines ‐ More doctors, nurses and other health workers ‐ Listening to patients more ‐ The quality of care should be improved ‐ No changes are needed
32	Views about the health system/what can be improved	Do you think the following areas of how health care is delivered by your GP and other doctors need improvement?	*Yes/No/Don't know to each of them* ‐ Improved communication among health professionals who look after me ‐ Better use of shared electronic records (such as MyHealth Record) ‐ More knowledgeable health professionals
33	Views about the system/views about specific types of services	How would you rate each of the following? ‐ The service offered by public hospitals ‐ The service offered by private hospitals ‐ Services provided by GPs and GP practices ‐ Services provided by dentists or and the services they offer ‐ Pharmacists or chemists and the services they offer ‐ Services provided by specialist doctors such as a cardiologist, psychiatrist, surgeon outside of hospitals in private practice ‐ Services provided by psychologists or counsellors ‐ Services provided by community care services delivered at home ‐ Residential aged care facilities including nursing homes and the services they provide ‐ Services provided by other allied health providers such as physiotherapists, dieticians and the services they offer	*Likert scale—1 (the service is very bad)‐5 (the service is excellent)*
34	Views about the system/confidence in accessing services	If you were to become seriously ill, how confident are you that you would… ‐ Get quality and safe medical care ‐ Receive the most effective medication ‐ Receive the best medical technology ‐ Be able to afford the care you need	*Likert scale‐ select one per item* ‐ Very confident ‐ Somewhat confident ‐ Not very confident ‐ Not all confident
35	Views about the health system/change over time—elective surgery	Compared to 10 y ago, do you think the amount of time that people in this country now have to wait for non‐emergency or elective surgery is longer, shorter or about the same? By non‐emergency or elective surgery we mean surgery for conditions that aren't immediately life threatening such as hip replacement or a cataract removal	*Select one* ‐ Longer ‐ About the same ‐ Shorter ‐ Don't know
36	Views about the health system/change over time—ability to get medical care	Compared to 10 y ago, is your ability to get the health care you need better, worse or about the same?	*Select one* ‐ Better ‐ About the same ‐ Worse ‐ Don't know
37	Views about the health system/change over time—ability to get medical care	In the next 10 y, do you think your ability to get the health care you need will get better, worse or about the same?	*Select one* ‐ Better ‐ About the same ‐ Worse ‐ Don't know
38	Private health insurance/value	What are all the reasons that you are covered by Private Health Insurance?	*[Noted only presented if ‘yes’ was selected to having PHI for Q8]* *Select as many as apply* ‐ Security/protection/peace of mind ‐ Life time cover/avoid age surcharge ‐ Choice of doctor ‐ Allows treatment as private patient ‐ Provides benefits for extras ‐ Shorter wait for treatment/concern over public hospital waiting lists ‐ Always had it/parents pay it/condition of job ‐ To gain government benefits/avoid extra Medicare levy ‐ Other financial reasons ‐ Has illness/condition that requires treatment ‐ Elderly/getting older/likely to need treatment ‐ Other
39	Private health insurance/value	What are all the reasons that you are not covered by private health insurance?	*Select as many as apply* ‐ Can't afford it/ too expensive ‐ High risk category ‐ Lack of value for money/not worth it ‐ Medicare cover sufficient ‐ Don't need medical care/in good health/have no dependants ‐ Won't pay Medicare and private health insurance premium ‐ Disillusioned about having to pay out‐of‐pocket costs or gap fees ‐ Prepared to pay costs of private treatment from own resources ‐ Pensioner/Veteran's affairs/ health concession card ‐ Not high priority or previously included in parents’ cover
